# Differential response to exercise in claudin-low breast cancer

**DOI:** 10.18632/oncotarget.21054

**Published:** 2017-09-19

**Authors:** Oliver K. Glass, Michelle Bowie, Julie Fuller, David Darr, Jerry Usary, Keara Boss, Kingshuk Roy Choudhury, Xioajing Liu, Zoe Zhang, Jason W. Locasale, Christina Williams, Mark W. Dewhirst, Lee W. Jones, Victoria Seewaldt

**Affiliations:** ^1^ Duke University Medical Center, Durham, NC, USA; ^2^ University of North Carolina at Chapel Hill, Chapel Hill, NC, USA; ^3^ Arrow Genomics LLC, Chapel Hill, NC, USA; ^4^ North Carolina State University, Raleigh, NC, USA; ^5^ Department of Medicine, Memorial Sloan Kettering Cancer Center, New York, NY, USA; ^6^ Weill Cornell Medical College, New York, NY, USA; ^7^ City of Hope Cancer Center, Duarte, CA, USA

**Keywords:** exercise, breast cancer, Hif1-α, mouse model, claudin-low

## Abstract

Exposure to exercise following a breast cancer diagnosis is associated with reductions in the risk of recurrence. However, it is not known whether breast cancers within the same molecular-intrinsic subtype respond differently to exercise. Syngeneic mouse models of claudin-low breast cancer (i.e., EO771, 4TO7, and C3(1)SV40Tag-p16-luc) were allocated to a uniform endurance exercise treatment dose (forced treadmill exercise) or sham-exercise (stationary treadmill). Compared to sham-controls, endurance exercise treatment differentially affected tumor growth rate: 1- slowed (EO771), 2- accelerated (C3(1)SV40Tag-p16-luc), or 3- was not affected (4TO7). Differential sensitivity of the three tumor lines to exercise was paralleled by effects on intratumoral Ki-67, Hif1-α, and metabolic programming. Inhibition of Hif1-α synthesis by the cardiac glycoside, digoxin, completely abrogated exercise-accelerated tumor growth in C3(1)SV40Tag-p16-luc. These results suggest that intratumoral Hif1-α expression is an important determinant of claudin-low breast cancer adaptation to exercise treatment.

## INTRODUCTION

Exposure to regular exercise after a diagnosis of primary breast cancer may lower the risk of recurrence and breast cancer death, compared with low exposure (inactivity) [1–3]. Intriguingly, recent epidemiological findings indicate that breast tumor sensitivity to exercise may differ as a function of clinical subtype with estrogen-receptor (ER) positive breast cancer being preferentially sensitive [4–6], although two independent cohort studies found triple negative tumors also to be responsive [7, 8]. Similar heterogeneity in response to exercise is observed in preclinical studies demonstrating that exercise inhibits primary breast tumor growth in some, but not all; both as a single modality [9, 10] and in combination with anticancer therapy [9, 11]. The underlying mechanisms that drive variation in exercise response in breast tumors are not currently defined.

To investigate whether breast tumors from the same molecular subtype exhibit a differential sensitivity to exercise treatment, we allocated three syngeneic mouse models of claudin-low breast cancer to a uniform endurance exercise-training dose. We show marked differential sensitivity of breast cancer to exercise treatment, which occurred with parallel changes to Hif1-α protein, and metabolism. Inhibition of Hif1-α synthesis abrogates growth stimulatory effects of exercise in one of the three tumor models. These results suggest that tumors adapt to exercise in different ways, and that part of this adaptation is Hif1 mediated.

## RESULTS

### Differential response of claudin-low breast cancer to exercise

To investigate whether breast tumors from the same molecular subtype exhibit a differential sensitivity to exercise treatment, female FVB/NJ, C57Bl/6J, and BALB/c mice (8-10 weeks of age) were randomized to endurance exercise training (treadmill running) or sham-control (stationary treadmill) groups. Mice randomized to exercise were acclimatized to treadmill running for 5.d for 10-15 mins/session at 10 m/min, followed by orthotopic implantation with EO771, 4TO7, or C3(1)SV40Tag-p16-luc (mammary fat pad; 1×10^5^ - 5×10^4^ -cells). Gene expression analysis showed that EO771, 4TO7 and C3(1)SV40Tag-p16-luc clustered to a classification of claudin-low (Supplementary Figure 1A, 1B). Following implantation endurance exercise was progressively increased to 20 m/min at 5% grade for 45 mins, 5 d.wk for 15 days; Mice randomized to sham-control groups were placed on a stationary treadmill for the same exposure time and frequency to standardize the environmental conditions. Body weights were not different across experimental groups for the duration of the study (Supplementary Figure 2A).

**Figure 1:**
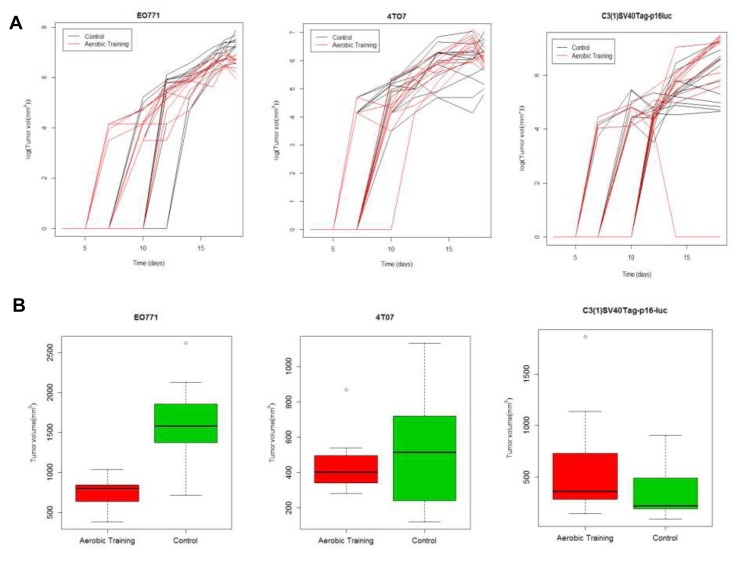
Aerobic training effects on breast tumor growth in mouse models of claudin-low breast cancer **A.** Aerobic training effects on tumor growth over time. (Left) EO771 mouse tumor model, individual mouse tumors. Values are log (tumor volume mm^3^) over time; (*n* = 12/group). (Middle) 4TO7 mouse tumor model, individual mouse tumors. Values are log (tumor volume mm^3^) over time; (*n* = 10-12/group). (Right) C3(1)SV40Tag-p16-luc mouse tumor model, individual mouse tumors. Values are log (tumor volume mm^3^) over time; (*n* = 10-12/group). **B.** Box-plots of final tumor volumes by group. (Left) EO771. Final volumes of mouse tumors at 18-days post tumor cell implantation by group; **p < 0.001; *p* = 1×10^-7^. (Middle) 4TO7. Final volumes of mouse tumors at 18-days post tumor cell implantation by group; *p* = 0.23. (Right) C3(1)SV40Tag-p16-luc. Final volumes of mouse tumors at 18-days post tumor cell implantation by group; *p < 0.05; *p* = 0.03.

At day 15, tumor volumes in exercise treatment groups were 0.5-fold smaller and 2-fold larger in EO771 (*p* < 0.001) and C3(1)SV40Tag-p16-luc (*p* = 0.03) tumors, respectively, compared with the control (Figure 1A, 1B; Supplementary Figure 2B; Supplementary Tables 1, 2). Tumor volumes at Day 15 were comparable between experimental groups in mice bearing 4TO7 tumors (*p* = 0.23). To ensure that differences in exercise treatment dose delivery between tumor models were not responsible for the observed differential tumor sensitivity to exercise, we measured citrate synthase (in the quadriceps femoris), an established marker of oxidative capacity and physiological adaptation to exercise [12]. Citrate synthase activity was not different across the groups (Supplementary Figure 2C), confirming exercise treatment dose was uniform.

**Figure 2:**
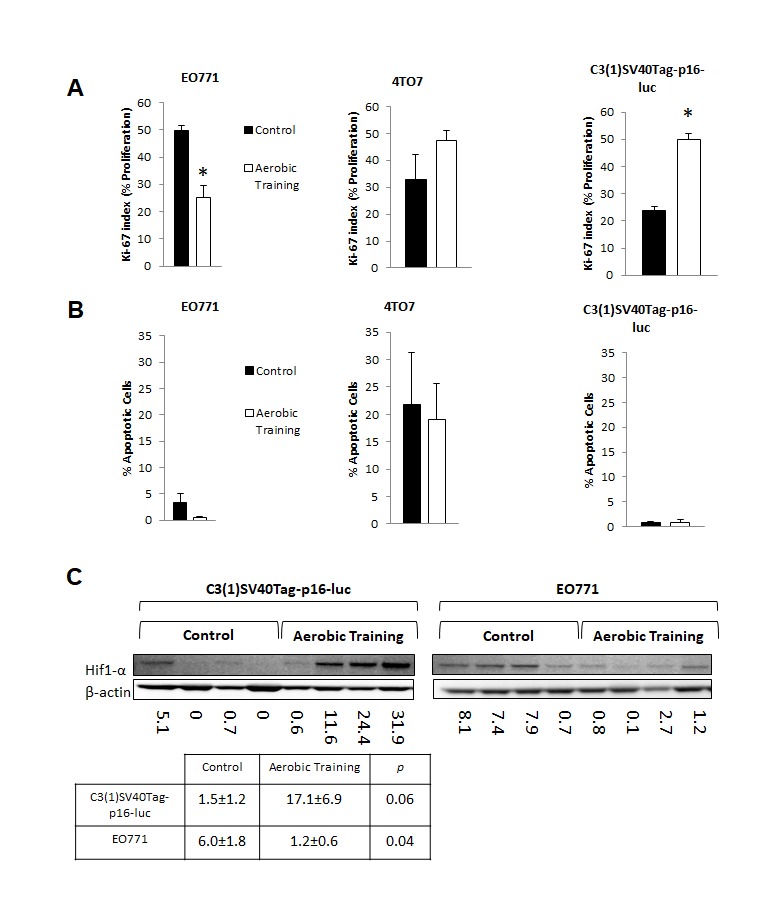
Aerobic training effects on proliferation, apoptosis, and Hif1-α in mouse breast tumors **A.** Effects of aerobic training on tumor cell proliferation compared to control by Ki-67 index. (Left) Proliferation of tumor cells in EO771 tumors, (Middle) Proliferation of tumor cells in 4TO7 tumors, (Right) Proliferation of tumor cells in C3(1)Tag-p16-luc tumors. Values are mean ± S.E.M. (*n* = 10-13/group); *p < 0.05. **B.** Effects of aerobic training on tumor cell apoptosis compared to control. (Left) Apoptosis of tumor cells within EO771 tumors, (Middle) Apoptosis of tumor cells within 4TO7 tumors, (Right) Apoptosis of tumor cells within C3(1)Tag-p16-luc tumors. Values are mean ± S.E.M. (*n* = 10-13/group). **C.** Western blot of Hif1-α in mouse tumors: Hif1-α western blot in C3(1)SV40Tag-p16-luc tumors (Bottom Left), EO771 tumors (Bottom Right) C3(1)SV40Tag-p16-luc mouse tumors: Mean densitometry normalized to β-actin : Aerobic Training, 17.11; Control, 1.45; (*p* = 0.06), EO771 mouse tumors: Mean densitometry normalized to β-actin : Aerobic Training, 6.02; Control, 1.19; (*p* = 0.04).

To determine whether differential tumor sensitivity to exercise correlated with changes in proliferation or apoptosis, we assessed Ki-67 and Cleaved Caspase-3, respectively, on Day 15. Compared to controls, the number of Ki-67-positive cells was 0.25-fold lower in EO771 (*p* < 0.001) and 1.26-fold higher in C3(1)SV40Tag-p16-luc (*p* = 0.001) in tumors of exercising animals (Figure 2A, Supplementary Figure 3). Ki-67 positive cells were comparable between groups in 4T07 (*p* = 0.28). No differences in Cleaved Caspase-3 positive cells were observed (Figure 2B, Supplementary Figure 4). Overall, these findings indicate differential sensitivity to exercise in breast tumors within the same molecular-intrinsic subtype is related to differences in proliferation.

### Hif1-α protein expression is a key regulator of exercise sensitivity

Hypoxia-inducible factor-1 (Hif1) is a key transcriptional regulator of aerobic and anaerobic metabolism [13]. We hypothesized that Hif1 plays a central role in controlling growth of tumors in response to exercise. In the C3(1)SV40Tag-p16-luc tumor Hif1 mediated alterations in tumor metabolism may provide an increase in macromolecular precursors for cell proliferation [14]. On the other hand, growth inhibitory effects of exercise on the E0771 tumor may be related to downregulation of Hif1 transcriptional activity. Hif1 activity is regulated by the oxygen sensitive sub-unit, Hif1-α. Under aerobic conditions, where Hif1 is inactive, Hif1-α is prolyl-hyroxylated and targeted for proteosomal degradation by the VHL complex. In normoxia, the relative lack of Hif1-α prevents heterodimerization and transcriptional activity [15] Hif1-α protein expression was 5-fold lower and 11-fold higher in EO771 and C3(1)SV40Tag-p16-luc, respectively, in tumors of exercising mice, compared with controls (Figure 2C). There were no differences in Hif1-α transcript levels between control and exercise treatment groups, indicating that the observed differences in Hif1-α protein level were not driven by differences in rate of synthesis. (Supplementary Figure 5B). This is to be expected, since the major point of regulation of Hif1-α is post-translational [15].

**Figure 3:**
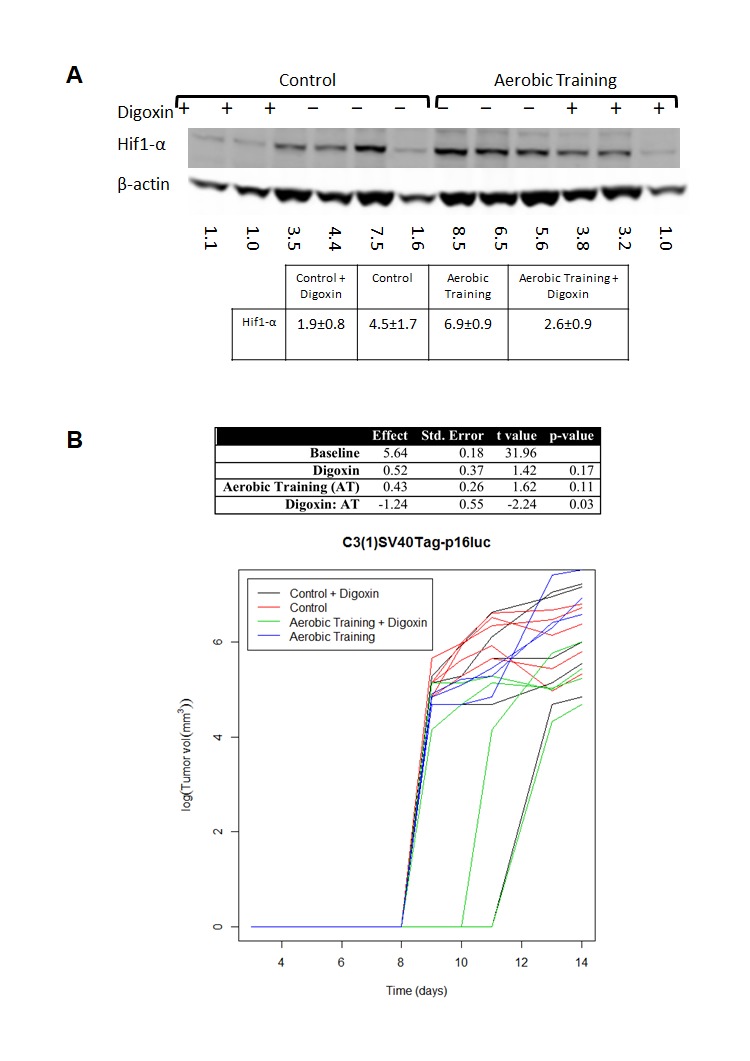
Aerobic training and Hif1-α inhibitor effects on mouse breast tumor growth **A.** Western blot of Hif1-α in mouse tumors treated with Digoxin (+) or Saline (-). Hif1-α western blot in C3(1)SV40Tag-p16-luc mouse tumors: Mean densitometry normalized to β-actin: Control plus Digoxin(+) 1.87; Control plus saline (-) 4.50; Aerobic Training plus saline (-) 6.87; Aerobic Training plus Digoxin (+) 2.58; *p* = 0.01 for Aerobic Training group with Digoxin compared to Aerobic Training plus saline, all other groups compared *p* > 0.05. **B.** C3(1)SV40Tag-p16-luc aerobic training tumor study ± Digoxin. Tumor bearing mice were treated 5 days/wk. with aerobic training or control ± Digoxin (2mg/kg) or equivalent volume saline based upon total bodyweight. Values are log (tumor volume) (*n* = 6/group); ANOVA analysis in table (Right).

To investigate the importance of Hif1-α in exercise-induced growth acceleration of C3(1)SV40Tag-p16-luc tumors, we used the cardiac glycoside, digoxin, to inhibit synthesis of Hif1-α protein [16, 17]. C3(1)SV40Tag-p16-luc bearing animals were randomly allocated to exercise or digoxin monotherapy (2 mg/kg) or combination therapy. Compared to sham-treated animals, intratumoral Hif1-α protein levels were lower in the combination treatment group (Figure 3A) and tumor sizes were not significantly different. These results showed that pharmacologic reduction of Hif1-α protein levels completely abrogated exercise-induced growth acceleration of C3(1)SV40Tag-p16-luc (Figure 3B; Supplementary Table 3).

### Exercise-induced changes in Hif1-α protein expression alters tumor metabolism

**Figure 4:**
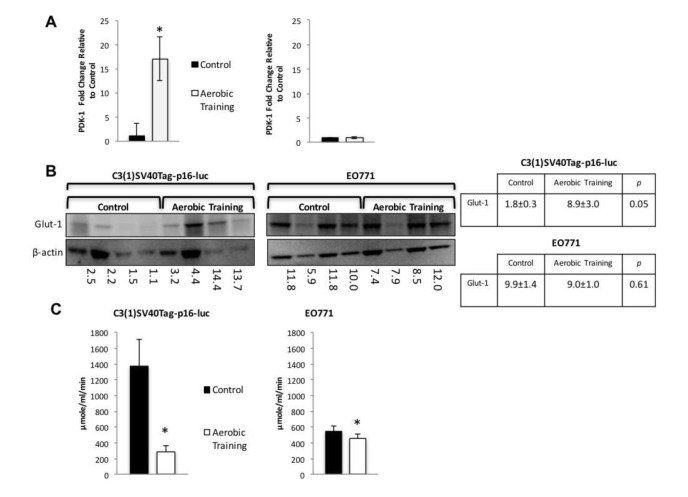
Aerobic training effects on PDK-1 gene expression, Glut-1 protein expression, and citrate synthase activity in mouse breast tumors **A.** PDK-1 gene expression in mouse tumors. PDK-1 gene expression shown as fold change relative to control. (Left) C3(1)SV40Tag-p16-luc on left; (Right) EO771. (*n* = 6/group). Values are mean ± S.E.M.; *p < 0.05. **B.** Glut-1western blot. C3(1)SV40Tag-p16-luc tumors (Left), EO771 tumors (Right); C3(1)SV40Tag-p16-luc mouse tumors: Mean densitometry normalized to β-actin : Aerobic Training, 8.92; Control, 1.84; (*p* = 0.05); EO771 mouse tumors: Mean densitometry normalized to β-actin : Aerobic Training, 8.97; Control, 9.91; (*p* = 0.61). **C.** Citrate synthase activity in mouse tumors. (Left) C3(1)SV40Tag-p16-luc control and aerobic training tumors, (Right) EO771 control and aerobic training tumors. Values are mean ± S.E.M. (*n* = 6/group); *p < 0.05.

Hif1 targets regulate glycolytic and reductive glutamine activity [18]. The Hif1 gene target, pyruvate dehydrogenase kinase-1 (PDK-1), inhibits pyruvate dehydrogenase (PDH), a potent regulator of mitochondrial oxidation of pyruvate [19]. Activation of PDK-1 increases glycolysis and reduces oxidative phosphorylation (OX PHOS) [20]. Therefore, we hypothesized that exercise would increase PDK-1 levels compared with controls in C3(1)SV40Tag-p16-luc. Compared to sham controls, exercise led to 17-fold higher expression of PDK-1 in C3(1)SV40Tag-p16-luc, with no differences in EO771 (Figure 4A). Similarly, expression of Glut-1 was 4.9-fold higher in C3(1)SV40Tag-p16-luc tumors after exercise treatment with no differences in EO771 (Figure 4B). These data suggest that differential Hif1-α protein expression led to Hif1-mediated changes in downstream protein targets involved in regulation of OX PHOS and glycolysis.

**Figure 5:**
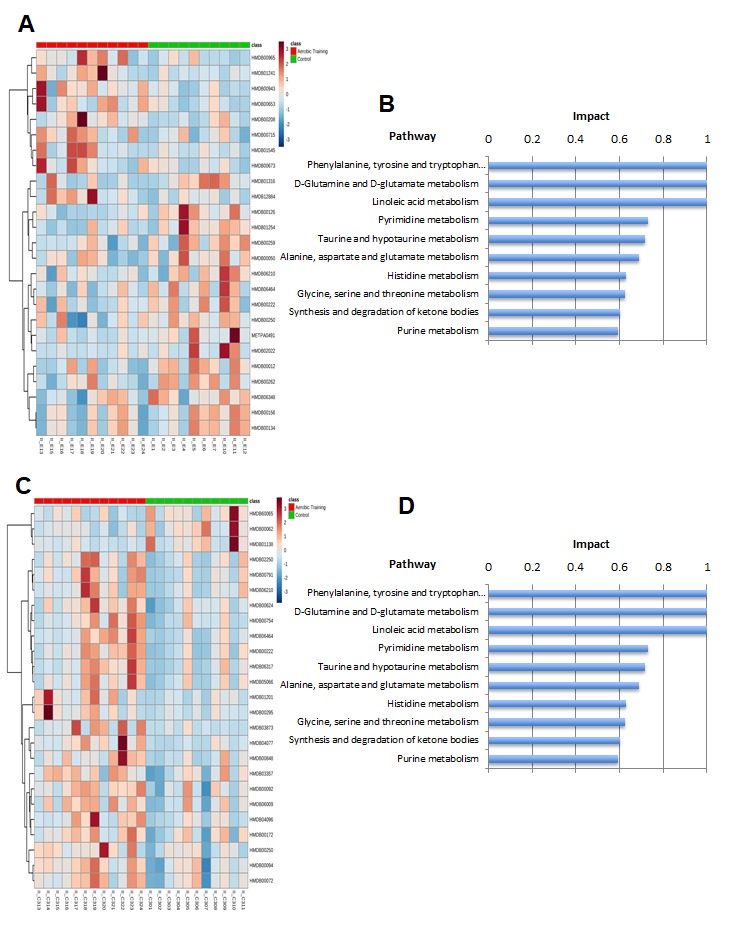
Metabolomic analysis of aerobic training effects on mouse breast tumors **A.** Hierarchical clustering heat map of top 20 metabolites in aerobic training group and control tumors in EO771 mouse model. **B.** Pathway impact analysis in EO771 mouse model in aerobic training group tumors compared to control. **C.** Hierarchical clustering heat map of top 20 metabolites in aerobic training group and control tumors in C3(1)SV40Tag-p16-luc mouse model. **D.** Pathway impact analysis in C3(1)SV40Tag-p16-luc mouse model in aerobic training group tumors compared to control.

We hypothesized that the differential sensitivity of intratumoral Hif1-α to exercise would be linked to alterations in tumor metabolism. We first assessed citrate synthase activity in tumors, as a marker of mitochondrial function and OX PHOS [21]. Citrate synthase expression was 1.2-fold lower (*p* = 0.01) and 4.7-fold higher in EO771 and C3(1)SV40Tag-p16-luc (*p* = 0.01), respectively in tumors of exercise treated mice, compared with controls (Figure 4C). These results suggested there are inherent differences in mitochondrial activity between these cell lines in response to exercise. Bioenergetic profiling revealed a higher basal (resting) extracellular acidification rate (ECAR) in C3(1)SV40Tag-p16-luc compared to EO771 tumor cells (*p* = 0.04) (Supplementary Figure 5C). Subsequent metabolomic analysis of pathway-associated metabolite sets showed that phenylacetate metabolism and the glycerol phosphate shuttle were lower in E0771 tumors after exercise treatment (Figure 5A, Supplementary Figure 6A) compared to control. These findings may partially explain exercise -induced inhibition of EO771 proliferation since glycerol-3-phosphate, and its corresponding acyltransferase, are positively correlated with proliferation in claudin-low breast cancer [22, 23]. Although the mechanisms remain to be fully elucidated, phenylacetate inhibits growth of several cancer cell lines [24]. Metabolic pathway impact analysis showed fatty acid, amino acid, and nucleotide metabolism were most affected in EO771 tumors from exercise treatment mice compared to control (Figure 5B).

**Figure 6:**
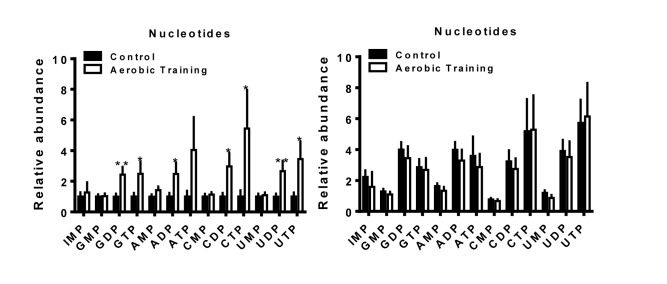
Aerobic training effects on intratumoral nucleotide metabolites Nucleotide metabolites in mouse tumors (Left) C3(1)SV40Tag-p16-luc; (Right) EO771. Relative abundance normalized to control; values are mean + S.E.M.; *p = 0.05-0.1, **p < 0.05.

The biosynthesis of amino acids, lipids, and nucleotides from substrates in the citric acid cycle are an important determinant of cell proliferation [25]. In C3(1)SV40Tag-p16-luc, metabolite sets of the citric acid cycle as well as the oxidation of long chain fatty acids (*p* < 0.05) were higher after exercise treatment, compared to sham-controls (Figure 5C, Supplementary Figure 6B, Supplementary Figure 7). ATP citrate lyase converts glucose derived acetyl-CoA to citrate that is essential for lipid synthesis; the source of acetyl-CoA is a HIF dependent process [18]. Metabolic pathway impact analysis indicated that exercise also affected fatty acid, amino acid, and nucleotide metabolism, but in the opposite direction from that seen with C3(1)SV40Tag-p16-luc (Figure 5D). Additionally, exercise led to higher levels of nucleotide metabolites in C3(1)SV40Tag-p16-luc (*p* < 0.05) (Figure 6). The presence of higher nucleotide metabolites is consistent with higher cell proliferation observed in C3(1)SV40Tag-p16-luc tumors of exercised mice [26].

Overall, our data suggest that exercise-induced differential modulation of Hif1-α and subsequent Hif1 transcriptional activity on tumor metabolism and cell proliferation, may underpin differential tumor sensitivity to exercise in claudin-low breast cancer.

## DISCUSSION

On the basis of observational studies, there is a growing consensus that exposure to exercise after a breast cancer diagnosis is associated with significant improvements in disease outcomes. However, there is a lack of consistency among risk reductions and breast subtypes [4–8]. Thus, identification of factors to predict or modulate tumor response to exercise is important, particularly given existence of exercise recommendations for cancer patients from several agencies [27, 28]. Here we find that modulation of Hif1-α expression plays a role in claudin-low breast cancer adaptation to exercise.

**Figure 7:**
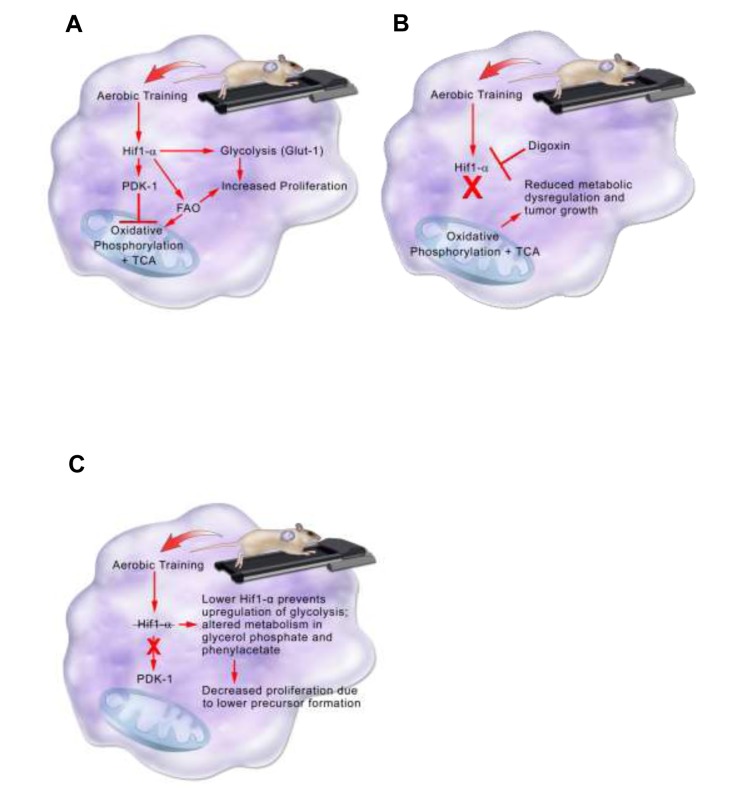
Model summarizing aerobic training effects on breast tumor biological pathways in the C3(1)SV40Tag-p16-luc model **A.** Aerobic training effects on breast tumor biological pathways in the C3(1)SV40Tag-p16-luc mouse model. Aerobic training in mice leads to stabilization of Hif1-α protein in C3(1)SV40Tag-p16-luc tumors. Hif1-α mediates inhibition of oxidative phosphorylation and citric acid cycle (TCA) through PDK-1 as well as upregulation of glycolysis through Glut-1. Hif1-α also increases fatty acid oxidation (FAO). Together increases in Glut-1 and FAO provide precursors for tumor cell proliferation. **B.** Aerobic training effects on breast tumor biological pathways with Hif1-α inhibition in the C3(1)SV40Tag-p16-luc mouse model. Hif1-α inhibition by the cardiac glycoside Digoxin prevents upregulation of glycolysis and inhibition of oxidative phosphorylation and TCA. Preventing Hif1-α metabolic reprogramming leads to reduced metabolic dysregulation and tumor growth. **C.** Model summarizing aerobic training effects on breast tumor biology in EO771 model. Aerobic training in mice decreases Hif1-α in EO771 tumors. Decreased Hif1-α protein alters metabolism in glycerol phosphate and phenylacetate, which may lead to decreased breast cancer cell proliferation through lower precursor formation.

In the C3(1)SV40Tag-p16-luc model, higher Hif1-α protein expression in tumors from exercised mice, contributed to acceleration of tumor growth (Figure 7). The importance of Hif1 in the adaptation to exercise was verified by inhibiting Hif1-α synthesis. Inhibition of Hif-1-α synthesis completely abrogated the accelerated growth associated with exercise. Metabolomic analysis in C3(1)SV40Tag-p16-luc was consistent with exercise-mediated expression of the Hif1 target, PDK-1. Higher PDK-1 expression was associated with increased Glut-1, reductive glutamine metabolism, and fatty acid synthesis. PDK-1 may be an important therapeutic target in claudin-low breast cancer to prevent metabolic reprogramming and inhibit accelerated tumor growth associated with exercise. Future studies evaluating exercise effects on other intratumoral Hif1 gene targets is warranted.

Interestingly, the exercise-induced acceleration of C3(1)SV40Tag-p16-luc is similar to prior work in p53 deficient MMTV-Wnt-1 [29]. Despite differences in tumorigenesis mechanism (transgenic p53^+/-^MMTV-Wnt-1; orthotopic C3(1)SV40Tag-p16-luc), both models suggest that exercise “stress” may drive adaptation to an aggressive phenotype. While the underlying mechanisms in p53^+/-^MMTV-Wnt-1 are unknown, exercise effects on p53 regulation of metabolism may have enhanced tumor growth [30]. p53, like Hif1, is a key mediator of cellular respiratory and glycolytic pathways. Since cross-talk exists between p53 and Hif1 [31], it is plausible that a metabolic shift towards increased glycolysis or reductive glutamine metabolism may be a common underlying mechanism for augmented tumor growth with exercise in the p53^+/-^MMTV-Wnt-1 and the C3(1)SV40Tag-p16-luc.

In contrast to C3(1)SV40Tag-p16-luc, exercise inhibited tumor growth in the EO771 model [9]. Here, exercise reduced Hif1-α levels and slowed tumor cell proliferation and tumor growth. Metabolomic data were diametrically opposed to that seen with C3(1)SV40Tag-p16-luc, indicating that this tumor was not able to adapt to exercise to support cellular proliferation and survival. The differences in responsiveness of C3(1)SV40Tag-p16-luc and E0771 to exercise may lie in their inherent differences in baseline metabolism. The C3(1)SV40Tag-p16-luc had a more glycolytic phenotype at baseline and may be able to more readily adapt to conditions of exercise than the E0771 tumor line.

The 4TO7 mouse model did not display tumor sensitivity to exercise. Tumor proliferation was comparable between groups suggesting that tumor growth in 4TO7 does not depend on Hif1. However, it is possible that our exercise prescription was insufficient to alter Hif1-α protein. Further interrogation into various exercise prescriptions and modalities in 4TO7 may help identify intratumoral targets that improve exercise sensitivity.

One limitation of our study is that we utilized citrate synthase activity as a surrogate measurement for the citric acid cycle and oxidative phosphorylation in tumors. Direct measurements of electron transport chain complexes provide a more accurate assessment of changes in oxidative phosphorylation. However, citrate synthase activity is commonly utilized to normalize oxidative phosphorylation enzymatic activities in isolated mitochondria, and thus may serve as an accurate surrogate marker [32]. Additionally, we did not directly measure hypoxia or perfusion in these experiments and can only speculate as to the role that differences in these physiologic parameters would have had on tumor growth and the Hif1-α signaling pathway.

Targeting breast cancer cellular metabolism may have clinical promise since actionable targets to treat triple-negative breast cancer, especially the claudin-low molecular intrinsic subtype, are lacking [33]. Many studies are investigating the uses of targeted inhibitors of key metabolic pathways to reverse metabolic dysregulation of breast tumors [34]. Our results suggest that exercise may be one effective strategy to alter claudin-low breast cancer metabolism. Furthermore, we demonstrate that exercise can modulate major cancer-intrinsic signaling pathways resulting in marked divergent growth promoting or inhibitory phenotypes. These data, by definition, challenge the longstanding rhetoric that exercise is a generic therapy that “works for everything” to one in which requires the adoption of a personalized medicine approach to fully interrogate the molecular mechanisms and predictors of response to optimize safety and efficacy.

## MATERIALS AND METHODS

### Cell culture

C3(1)SV40Tag-p16-luc breast cancer cells that stably express luciferase were kindly provided by the lab of Dr. Ned Sharpless and the UNC Mouse Phase 1 Unit, University of North Carolina. C3(1)SV40Tag-p16-luc cells were isolated from the tumor of a C3(1)SV40Tag transgenic mouse and transfected with a stable expressing p16 luciferase reporter. C3(1)SV40Tag-p16-luc cells were cultured in DMEM (High glucose, CellGro) supplemented with 10% fetal bovine serum, antibiotic-antimycotic (Life technologies), and 0.2mg/kg G418/Geneticin. EO771 breast cancer cells were cultured in RPMI-1640 with L-glutamine, 8% fetal bovine serum, sodium pyruvate (Life Technologies), and MEM non-essential amino acids (Life technologies). 4TO7 cells were grown in DMEM supplemented with 10% fetal bovine serum and antibiotic-antimycotic (Life Technologies). All cells were trypsinized at 80% confluence in log phase growth.

### Mouse cell line breast cancer molecular subtyping

C3(1)SV40Tag-p16-luc, EO771, and 4TO7 cell lines were subjected to micro-array and cluster analysis by the University of North Carolina Mouse Phase I Unit to determine specific breast cancer subtype; all cells lines clustered with the claudin-low breast cancer subtype (data not shown). Microarray analysis was performed as previously described [35]. Briefly, total RNA was purified from 20-30 mg of mouse mammary tumor using a Qiagen (Valencia, CA USA) RNeasy Mini Kit following the manufacture's protocols. RNA quantity was determined using a Nanodrop spectrophotometer. An Agilent Low RNA Input Fluorescent Linear Amplification Kit was used and total tumor RNA was reverse transcribed and labeled with cyanine-5 (Cy5) dye for experimental samples. Whole mouse total RNA was labeled with cyanine-3 (Cy3) dye and used for the reference samples. Equal quantities of the labeled mouse reference RNA and tumor RNA were hybridized overnight to Agilent 4×180K microarrays. They were then washed, scanned, and their signal intensities were determined. Microarray data was stored in and extracted from the UNC Microarray Database as log2 Cy5/Cy3 ratios. The values for each probe were Lowess normalized and those with intensity values greater than 10 in both channels were selected then the median expression value was calculated for each probe. Hierarchical clustering was performed with the unsupervised data set using centroid linkage and was then viewed using Java Treeview [36].

### Mice

7-9 week old female mice (FVB/NJ, BALB/c, C57/Bl6) were obtained from Jackson Labs and maintained on a reverse 12:12 hour light-dark cycle in a low-stress environment. Animals were group housed ~4-5 mice/cage and provided food and water *ad libitum*. Animal care and all experimental procedures were in accordance with the Institutional Animal Care and Use Guidelines at Duke University Medical Center.

### Orthotopic tumor cell implantation

Cell lines were trypsinized at 80% confluence and resuspended 1:1 in Hank's Balanced Salt Solution (HBSS) (Life technologies) or Phosphate-Buffered Saline (PBS) (Life technologies) in matrigel (BD Biosciences). Phenol-red free matrigel was used for EO771 cells as data by our own group suggest they are estrogen receptor (ER) responsive (data not shown). Mice were anesthetized using intraperitoneal injections of ketamine (8.5 mg/kg) and xylazine (8.5 mg/kg). Prior to tumor cells implantation, mouse hair was removed around area of injection site with Nair™ (Church & Dwight Co., Inc., Ewing, NJ) to allow for improved accuracy of surgical implantation of tumor cells and enhanced tumor volume measurements. Following hair removal, the surgical field was prepared using three alternating applications of chlorohexidine gluconate scrub solution and 70% ethanol. Using a sterile scalpel, a skin incision was made exposing the dorsal mammary fat pad and 5 × 10^4^ C3(1)SV40Tag-p16-luc cells were injected in 0.1ml of 1:1 HBSS and Phenol-red containing matrigel per FVB/NJ female mouse; 1 × 10^5^ EO771 cells were injected in 0.1ml of 1:1 PBS and Phenol-red free matrigel per C57/Bl6 female mouse; 2.5 × 10^5^ 4TO7 cells were injected in 0.1ml of 1:1 PBS and Phenol-red containing matrigel per BALB/c female mouse. The number of cells injected was determined in a prior pilot study to obtain the minimum number of cells to form breast tumors and allow for consistent number of days of aerobic training.

### Aerobic training intervention

Mice were assigned to either aerobic training (*n* = 12) or control (*n* = 12) treatment. Control mice were placed on stationary treadmills (Columbus Instruments, Columbus, OH) in the same room as the aerobic training group during training sessions to maintain identical environmental conditions for all mice. Stationary treadmills were powered on, but the treadmill belt remained idle during the intervention. Aerobic training sessions were performed at the beginning of the active dark cycle in order to more accurately assess normal murine physiologic tendencies towards physical activity. The aerobic training group participated in an acclimatization period prior to study initiation. The acclimatization period on the treadmill (Columbus Instruments, Columbus, OH) apparatus consisted of the following: 5 minutes at 5m/min. at 5% grade on pre-study Day 1; pre-study Days 2-4 speed was increased by 5m/min. each day for 5 additional minutes until a final dose was achieved of 20m/min. for 5 minutes on pre-study Day 4. Pre-study Day 5, control and aerobic training groups of mice were injected with breast cancer cell lines; this date was considered study Day 1. On study Day 3, the aerobic training intervention was initiated. Mice in the aerobic training group were treated 5 consecutive days/wk. using the following aerobic training doses: 5m/min for 5min., 10m/min. for 5min., 15m/min. for 5 min., and 20m/min. for 45 min., which is equivalent to 70% VO_2peak_ as previously described [37]. Total aerobic training time per session was 60 minutes with an average overall distance completed of 1,055m.

### Digoxin and saline treatment in mice

Following aerobic training using the protocol previously described or control (placement of mice on a stationary treadmills), mice were injected intraperitoneally (i.p.) with either digoxin at 2mg/kg or equivalent volume of saline for corresponding bodyweight using 1ml syringes. Digoxin was purchased from Duke University Hospital Pharmacy as 250 mcg/1ml ampules. Mice (*n* = 6/group) were injected 5 days/week immediately following the exercise prescription or control.

### Breast tumor measurement and body weight

Mouse tumors and body weights were measured three times per week using digital calipers and a digital scale respectively. Length and width measurements in millimeters (mm) were recorded and tumor volume was calculated using ((l x w^2^)/2).

### Sacrifice and tissue collection

All animals were sacrificed at ~18 days post breast cancer cell line injection and 24 hr. following the last aerobic training session using an intraperitoneal (i.p.) injection of 0.05ml Euthasol^®^. Tumors were surgically removed and measured using calipers to determine final tumor volume. Additionally, tumors were weighed to investigate differences in tumor mass and hearts were weighed to examine cardiomyopathy as a result of physiologic adaptation to aerobic training. Muscle was excised from the quadricep femoris to investigate muscle adaptations to aerobic training stimulus. Tumor and muscle tissue were flash frozen in liquid nitrogen and stored at -80°C until analysis. A portion (~40%) of tumor tissue was fixed in formalin and stored at 4°C in 70% ETOH.

### Citrate synthase activity

-80°C stored frozen mouse quadricep femoris and tumor tissue (~25mg) were homogenized at 4°C with a motorized tissue homogenizer for ~3s cycles in MT Cell Lysis Solution (Sigma-Aldrich) supplemented with protease inhibitor cocktail solution at 1:10 dilution (Roche) and 1x phosphatase inhibitor cocktail solution (Sigma-Aldrich). Homogenized tissue solution was then spun at 14,000 rpm at 4°C for 10 min. and supernatant was collected. BCA protein assay (Thermo Scientific) was then performed according to the manufacturer protocol, and protein concentration was determined against BSA standards from an absorption wavelength of 550nm on a BioRad 680 Plate Reader. 8μg of total protein lysate per sample was used in accordance with the Citrate Synthase Assay Kit (Sigma-Aldrich). Samples were run in triplicate on a 96-well microplate. Maximal velocity measurements were recorded at 415nm in 10-second intervals for 90 seconds. Net citrate synthase activity was determined from the equation provided in the Sigma Aldrich Citrate Synthase Assay protocol manual.

### Seahorse bioenergetic analysis

The XF24 Analyzer (Seahorse Biosciences, North Billerica MA) was used to measure the bioenergetic profile of C3(1)SV40Tag-p16-luc and E0771 cell lines. The XF24 analyzer allows for real-time measurements of extracellular acidification rate (ECAR). Mitochondrial function was determined in the presence of mitochondrial uncoupling drugs Oligomycin, FCCP and antimycin. A; these were injected sequentially through ports in the XF Assay cartridges to final concentrations of 1 μg/ml, 1 μM and 10 μM respectively. This allowed for the determination of baseline mitochondrial activity, energy consumption from ATP production, and maximal respiration.

Cells are plated into the XF24 cell culture plate (Agilent Technologies) at a density of 60,000 cells per well, 5 wells per cell line. This is a density previously determined to produce a confluent plate the following morning. Cells were incubated overnight in regular growth medium to allow for attachment. 1 hour prior to assay initiation, the growth media was removed for DMEM (Agilent Technologies) supplemented with 0.18% glucose (45% D-(+)-glucose, Sigma), sodium pyruvate (Thermo), and glutamax (Thermo). At run completion, BCA assay provided total protein per well per cell line, confirming that variation in cell density was not a contributing factor to the observed effects. Data was averaged for each cell line. Statistical analysis was performed *via* 2-way ANOVA using the Graphpad PRISM software.

### RT-PCR

RNA was extracted from frozen ~25 mg sections of tumors using the Qiagen RNeasy Miniprep Kit (Qiagen, Inc, Valencia, CA) according to the manufacturer's protocol. One μg of RNA was reverse-transcribed using the Bio-Rad iScript cDNA Synthesis Kit (Bio-Rad Laboratories, Inc, Hercules, CA), and resulting cDNA was stored at -20°C until analysis. Quantitative Real-Time PCR (QPCR) was performed using SsoAdvanced Universal SYBRGreen Supermix (Bio-Rad) on a Roche LightCycler 480 (Roche): Polymerase Activation and Denaturation, 30s at 95°C; 35 cycles of 10s at 95°C (Denaturation), 20s at 60°C (Annealing/Extension); 65°C-95°C, 0.5°C increment 2 sec/step (Melt Curve Analysis). Gene expression was quantified (2^-ΔΔCp^) relative to the β-actin housekeeping gene. All primers were purchased and validated from Bio-Rad using Bio-Rad pre-selected target sequences: β-actin qMmuCED0027505; Hif1-α qMmuCID0005501; Pdk-1 qMmuCED0044540.

### Western blot

Protein lysates were obtained using the methods described in the Citrate Synthase Activity methods sub-section. Denatured protein lysates were subjected to a NuPAGE Novex 4-12% Bis-Tris protein gel, transferred to membranes, and blocked in 5% milk/Tris-buffered saline with 0.1% Tween-20 (TBST) or 5% BSA/Tris-buffered saline with 0.1% Tween-20 for one hour. After washing with TBST, membranes were incubated with primary antibodies in 5% BSA/TBST overnight at 4°C. Primary antibodies were used at the following dilutions: anti-Hif1-α (CS-3716) Cell Signaling Technology (Beverly, MA) 1:500, anti-phospho-p70S6Kinase (CS-9234) Cell Signaling Technology (Beverly, MA) 1:500, anti-β-actin (SC-47778) Santa Cruz Biotechnology (Santa Cruz, CA) 1:4000, anti-phospho-Akt (CS-4060) Cell Signaling Technology (Beverly, MA) 1:1000, anti-Glut-1 (ab-652) Abcam (Cambridge, MA) 1:1000. After washing with TBST, membranes were incubated with peroxidase-conjugated donkey anti-rabbit secondary antibody (SC-2313) Santa Cruz Biotechnology (Santa Cruz, CA) at 1:20,000 in 1%milk/TBST for 1.5 hours at room temperature. After washing with TBST, blots were visualized with SuperSignal West Pico Chemiluminescent Substrate (Thermo Scientific; Rockford IL) on a Kodak Image Station 2000mm. Densitometric analysis was performed using Carestream Molecular Imaging software v. 5.0.2.30 (Carestream Health, Inc.).

### IHC

#### Ki67

Paraffin embedded sections were deparaffinized and rehydrated through a xylene, 100% ETOH, 95% ETOH, dH_2_O gradient. Slides were then washed in dH_2_O and antigen was unmasked in boiling 10mM sodium citrate pH 6.0 for 10 minutes. Slides were incubated in 3% hydrogen peroxide and each slide was blocked in 5% Goat Serum/TBST for one hour. Primary Ki-67 antibody (CS-12202) Cell Signaling Technology (Beverly, MA) was diluted 1:400 in SignalStain Antibody Diluent Cell Signaling Technology (Beverly, MA) and incubated overnight at 4°C. Slides were washed with TBST and covered with three drops of SignalStain Boost Detection Reagent (CS-8114) Cell Signaling Technology (Beverly, MA). After washing with TBST slides were stained with SignalStain DAB Substrate kit (CS-8059) Cell Signaling Technolgy (Beverly, MA), counterstained with hematoxylin, dehydrated, and coverslipped.

### Cleaved caspase-3

Paraffin embedded sections were deparaffinized and rehydrated through a xylene, 100% ETOH, 95% ETOH, dH_2_O gradient. Slides were then washed in dH_2_O and antigen was unmasked in boiling 10mM sodium citrate pH 6.0 for 10 minutes. Slides were incubated in 3% hydrogen peroxide and each slide was blocked in 5% Goat Serum/TBST for one hour. Primary Cleaved Caspase-3 antibody (CS-9579) Cell Signaling Technology (Beverly, MA) was diluted 1:500 in SignalStain Antibody Diluent Cell Signaling Technology (Beverly, MA) and incubated overnight at 4°C. As a negative control Rabbit isotype (CS-3900) Cell Signaling Technology (Beverly, MA) was used at 1:500 dilution. Slides were washed with TBST and covered with three drops of SignalStain Boost Detection Reagent (CS-8114) Cell Signaling Technology (Beverly, MA). After washing with TBST slides were stained with SignalStain DAB Substrate kit (CS-8059) Cell Signaling Technolgy (Beverly, MA), counterstained with hematoxylin, dehydrated, and coverslipped.

### Microscopy and image analysis

Briefly, Ki67 and Cleaved Caspase-3 slides were captured on an Applied Biosystems Arcturus LCM System using Arcturus XT software (Life Technologies, Grand Island, NY). Image J software (NIH, http://imagej.nih.gov/ij/) was used to manually count cells for Ki-67 and Cleaved Caspase-3.

### Metabolomics

#### Metabolite extraction in tumor

Mouse tumors were extracted the same way as described previously [38]. Briefly, the tumor sample was first homogenized in liquid nitrogen and then 5 to 10 mg was weighed in a new Eppendorf tube. Ice cold extraction solvent (250 μl) was added to tissue sample, and a pellet mixer was used to further break down the tissue chunk and form an even suspension. After incubation on ice for an additional 10 min, the tissue extract was centrifuged with the speed of 20 000 g at 4 °C for 10 min. The supernatant containing 1.5 mg tissue extract was transferred to a new Eppendorf tube and dried in vacuum concentrator. The dry pellets were reconstituted into 30 μl sample solvent (water:methanol:acetonitrile, 2:1:1, v/v) and further analyzed by LC-high resolution mass spectrometer (HRMS).

### LC-HRMS

Ultimate 3000 UHPLC (Dionex) is coupled to Q Exactive Plus-Mass spectrometer (QE-MS, Thermo Scientific) for metabolite separation and detection. For polar metabolite analysis, a hydrophilic interaction chromatography method (HILIC) with an Xbridge amide column (100 × 2.1 mm i.d., 3.5 μm; Waters) is used for compound separation at room temperature. The instrument condition, mobile phase, gradient information and metabolomics data analysis were described previously except that mobile phase A was replaced with water containing 5 mM ammonium acetate (pH 6.8) [39].

### Statistical analysis

Statistical analysis was carried out using SAS University Edition (SAS Institute, Inc, Cary, NC). Descriptive statistics are presented as mean ± SEM. One-way ANOVA was used to compare differences in tumor variables between groups. Differences in apoptosis, proliferation, gene expression, citrate synthase activity and, densitometric values were evaluated using *t*-tests. For all tests, *p* < 0.05 was considered statistically significant.

### Analysis of exercise effect on tumor growth rate

We fit a model of the form:

$$\text{log }y_{ij}\left(t\right)\;=\;\mu \;+\;b_{i}\;+\;{\text{α}}_{j}\;+\;\beta t\;+\;{\text{β}}_{j}t\;+\;\varepsilon_{ijt}$$ (0.1)

Where *y*_ij_(*t*) is the tumor volume at time *t* for the i-th animal in the *j*-th group (Control or Aerobic Exercise). Model (0.1) explains variation in tumor volumes in terms of an initial baseline volume μ, representing the Control group at time 0, an effect for the aerobic exercise on the initial tumor volume *α*_j_, an effect for the *i*-th animal on the initial tumor volume *b*_i_, assumed to have a zero mean Gaussian distribution, a linear growth rate for the baseline group β and an effect of aerobic exercise on the growth rate *β*_j_. Finally *ε*_ijt_ is measurement error, also assumed to have a zero mean Gaussian distribution, independent of *b*_i_. Model was fit to the data using the method of restricted maximum likelihood using the nlme package in the R computing platform (www.r-project.org).

### Metabolomics analysis

Using MetaboAnalyst 3.0 (www.metaboanalyst.ca) pathway analysis was performed using HMDB ID, without data filtering, normalization, transformation, or scaling. The parameters for the pathway impact analysis were pathway-associated metabolite sets in mice using all of the compounds in the set library. Statistical analysis was performed using unpaired sample concentrations, without data filtering, normalization, transformation, or scaling. Hierarchical clustering was executed using heat map with the following settings: detailed view, samples not reorganized, top 20, showing cell borders.

## SUPPLEMENTARY MATERIALS FIGURES AND TABLES



## Abbreviations

ER: Estrogen Receptor
Hif1: Hypoxia Inducible Factor-1
PDK-1: Pyruvate Dehydrogenase Kinase-1
PDH: Pyruvate Dehydrogenase
OXPHOS: Oxidative Phosphorylation
ECAR: Extracellular Acidification Rate
