# Supplementary material for: Differential response to exercise in claudin-low breast cancer
Source: Oncotarget. 2017 Sep 19;8(60):100989–1004. doi: 10.18632/oncotarget.21054 (PMC5731850; doi:10.18632/oncotarget.21054)
Supplement: Supplementary file 1 [file oncotarget-08-100989-s001.pdf]

Differential response to exercise in claudin-low breast cancer

SUPPLEMENTARY MATERIALS

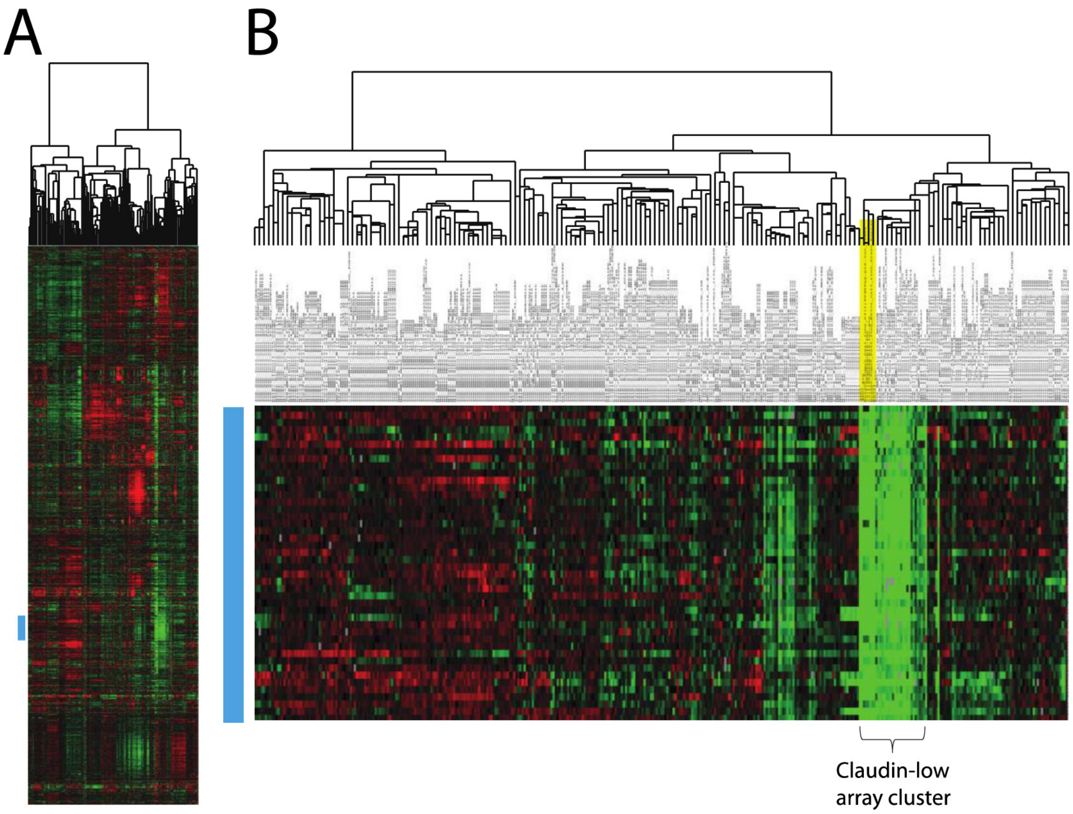

Supplementary Figure 1: Microarray of C3(1)SV40Tag-p16-luc, EO771, and 4TO7 cell lines. A. 1,855 intrinsic genes were used to hierarchical cluster 393 Agilent microarrays. The subset of the cluster with the claudin-low genes is highlighted with a blue bar on the left. **B**. A close-up of the claudin-low gene section shows the claudin-low array sub-cluster that includes 4TO7, C3(1)SV40Tag-p16-luc, and EO771 cell-line arrays highlighted in gold.

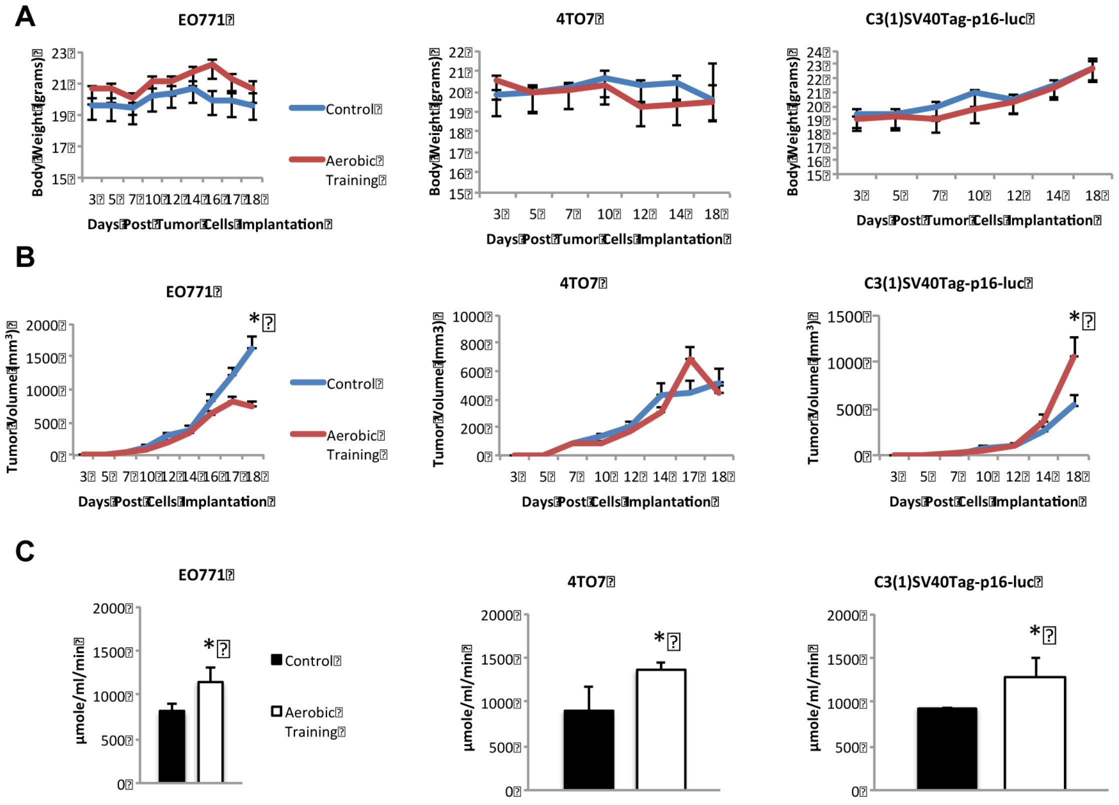

Supplementary Figure 2: Aerobic training effects on bodyweight measurements, absolute tumor volumes, and intramuscular citrate synthase activity in mouse models of claudin-low breast cancer. **A**. Mouse bodyweight over time. (Left) EO771; (Middle) 4TO7; (Right) C3(1)SV40Tag-p16-luc. Values are presented as mean ± S.E.M. (n=10-12/group). **B**. Mean tumor volumes over time. (Left) EO771; (Middle) 4TO7; (Right) C3(1)SV40Tag-p16-luc. Values are presented as mean ± S.E.M. (n=10-12/group); *$p<0.05$. **C**. Citrate synthase activity in mouse quadriceps femoris following aerobic training or control treatment. (Left) EO771, $p=0.03$; (Middle) 4TO7, $p=0.04$; (Right) C3(1)SV40Tag-p16-luc, $p=0.03$. Values are presented as mean ± S.E.M. (n=6/group); *$p<0.05$.

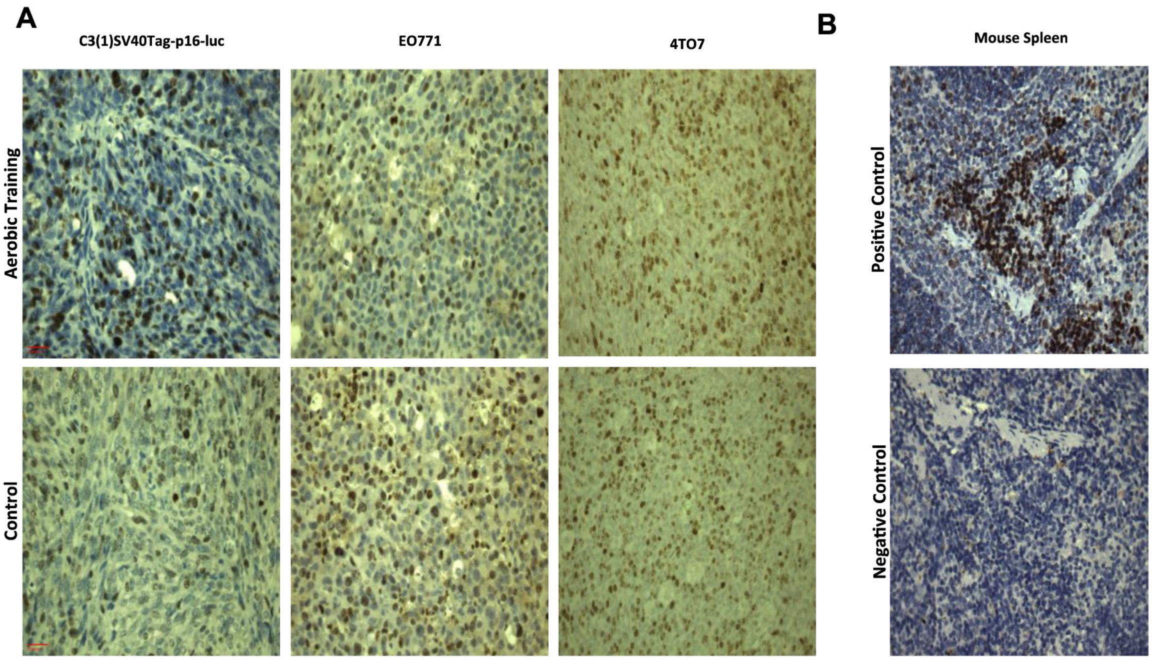

Supplementary Figure 3: Ki-67 stained tumor sections from C3(1)SV40Tag-p16-luc and EO771 mouse tumors. **A**. (Top left) Typical C3(1)SV40Tag-p16-luc tumor section from aerobic training group tumor counterstained with hematoxylin; (Top middle) typical EO771 tumor section from aerobic training group tumor counterstained with hematoxylin; (Top right) typical 4TO7 tumor section from aerobic training group tumor counterstained with hematoxylin; (Bottom left) typical C3(1)SV40Tag-p16-luc tumor section from control group tumor counterstained with hematoxylin; (Bottom middle) typical EO771 tumor section from control group tumor counterstained with hematoxylin; (Bottom right) typical 4TO7 tumor section from control group tumor counterstained with hematoxylin. **B**. Controls for Ki-67 staining. (Top) Mouse spleen section as positive control; (Bottom) no primary antibody negative control.

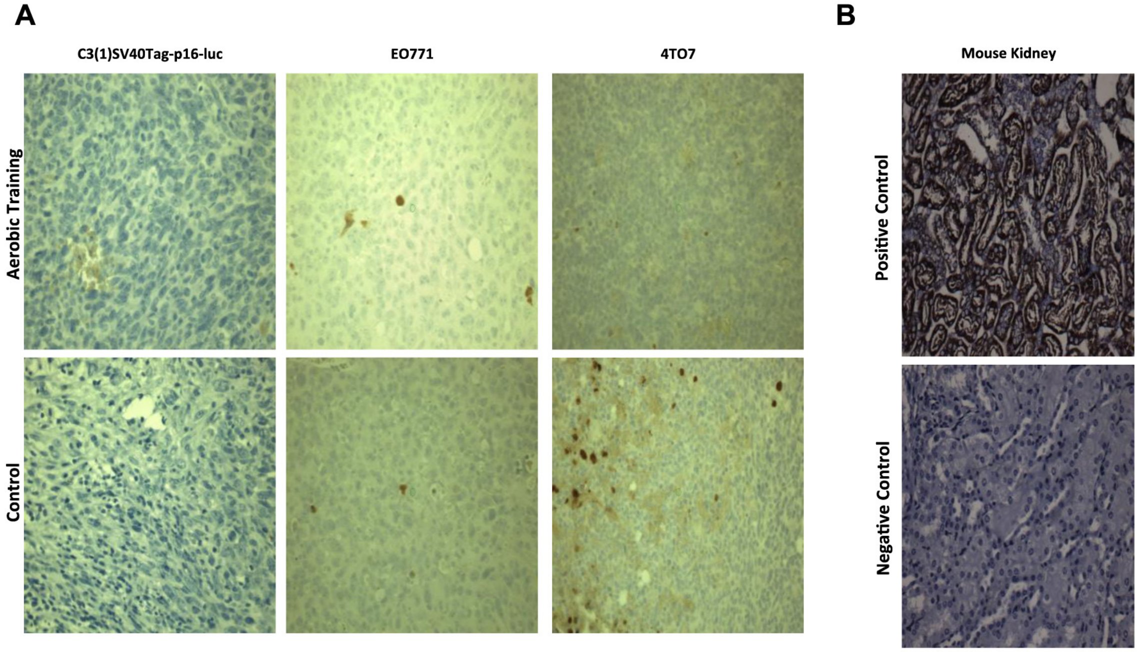

Supplementary Figure 4: TUNEL staining of tumor sections from C3(1)SV40Tag-p16-luc and EO771 mouse breast tumors. **A**. (Top left) Typical C3(1)SV40Tag-p16-luc tumor section from aerobic training group tumor counterstained with hematoxylin; (Top middle) typical EO771 tumor section from aerobic training group tumor counterstained with hematoxylin; (Top right) typical 4TO7 tumor section from aerobic training group tumor counterstained with hematoxylin; (Bottom left) typical C3(1)SV40Tag-p16-luc tumor section from control group tumor counterstained with hematoxylin; (Bottom middle) typical EO771 tumor section from control group tumor counterstained with hematoxylin; (Bottom right) typical 4TO7 tumor section from control group tumor counterstained with hematoxylin. **B**. Controls for TUNEL staining. (Top) Mouse kidney positive control; (Bottom) no primary antibody negative control for nonspecific staining.

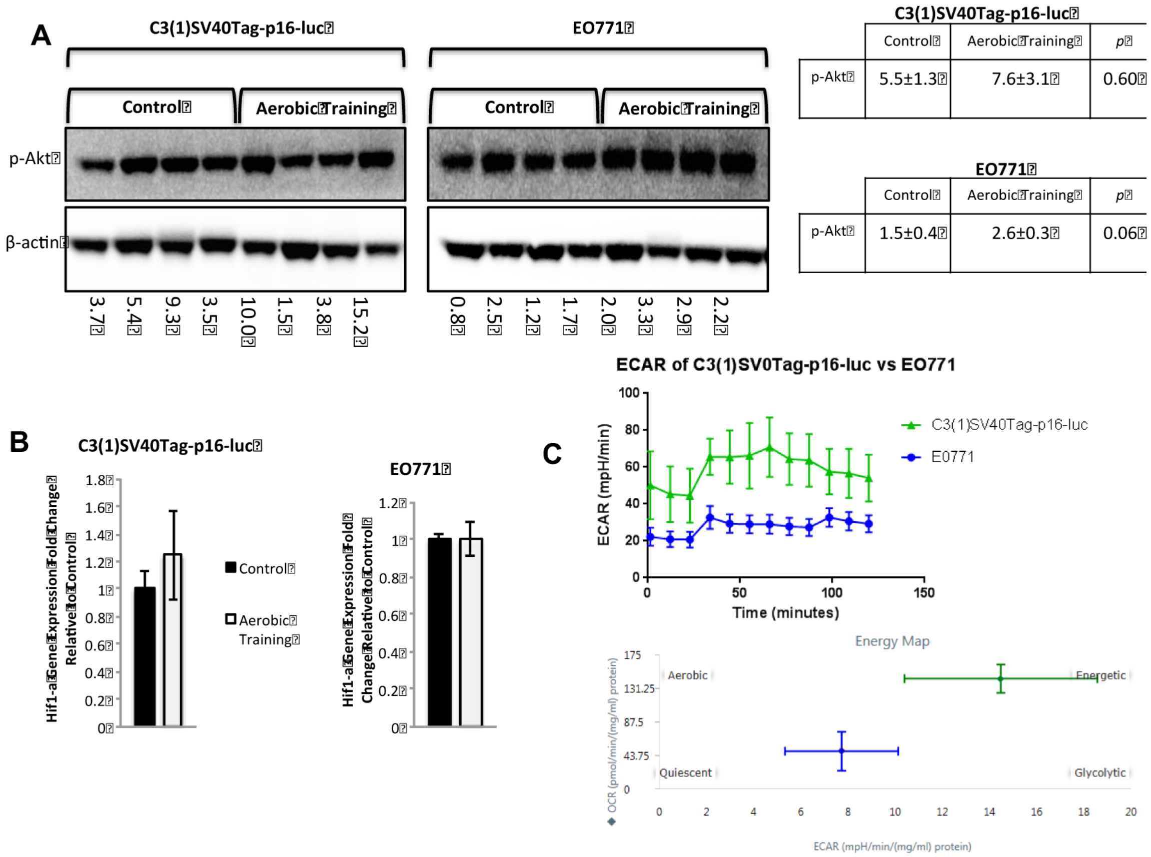

Supplementary Figure 5: Phospho-Akt western blot and Hif1-α gene expression in C3(1)SV40Tag-p16-luc and EO771 mouse breast tumors; Seahorse extracellular acidification rate and bioenergetics profile in C3(1)SV40Tag-p16-luc and EO771 cell lines. **A**. Western blots of phospho-Akt in mouse tumors: Phospho-Akt western blot in C3(1)SV40Tag-p16-luc tumors (Top Left), EO771 tumors (Top Right); C3(1)SV40Tag-p16-luc mouse tumors: Mean densitometry normalized to β-actin: Aerobic training, 7.6; control, 5.5; (p=0.60); EO771 mouse tumors: Mean densitometry normalized to β-actin: Aerobic training, 2.6; control, 0.3; (p=0.06). Values are presented in the chart mean ± S.E.M. **B**. Hif1-α gene expression in mouse tumors. Hif1-α gene expression relative to control. Values indicate fold change and are mean ± S.E.M.; (n=6/group). **C**. Seahorse extracellular acidification rate and bioenergetics profile in cell lines. (Top) Extracellular acidification rate (ECAR) over time for C3(1)SV40Tag-p16-luc and EO771 cell lines (p=0.04). Values are mean ± S.E.M.; (n=5/cell lines); *p<0.05. (Bottom) Bioenergetic map of C3(1)SV40Tag-p16-luc and EO771 cell lines presented as oxygen consumption rate (OCR) vs. ECAR.

A

HMDB ID	p.value	Name
HMDB00126	0.010724	Glycerol 3-phosphate
HMDB01254	0.019055	Glucosamine 6-phosphate
HMDB00012	0.022156	Deoxyuridine
HMDB00259	0.02374	Serotonin
HMDB00943	0.026423	Threonic acid
HMDB00965	0.046176	Hypotaurine
HMDB06464	0.046735	Elaidic carnitine
HMDB00653	0.049994	Cholesterol sulfate

B

HMDB ID	p.value	Name
HMDB06464	0.00357	elaidic carnitine
HMDB00222	0.0053223	L-Palmitoylcarnitine
HMDB00094	0.0080393	Citric acid
HMDB00624	0.008836	D-Leucic acid
HMDB04077	0.011031	4,6-Dihydroxyquinoline
HMDB06317	0.017079	trans-Hexadec-2-enoyl carnitine
HMDB00791	0.017654	L-Octanoylcarnitine
HMDB03873	0.01892	3a,7a,12a-Trihydroxy-5b-cholestanoic acid
HMDB00754	0.019282	3-Hydroxyisovaleric acid
HMDB01201	0.020997	Guanosine diphosphate
HMDB00072	0.022145	cis-Aconitic acid
HMDB00092	0.022781	Dimethylglycine
HMDB05066	0.024133	Tetradecanoylcarnitine
HMDB04096	0.025156	5-Methoxyindoleacetate
HMDB00062	0.026579	L-Carnitine
HMDB00250	0.028559	Pyrophosphate
HMDB06009	0.029625	Isoputreanine
HMDB00295	0.036899	Uridine 5'-diphosphate
HMDB03357	0.039887	N-Acetylornithine
HMDB00848	0.040365	Stearoylcarnitine
HMDB02250	0.040572	Dodecanoylcarnitine
HMDB06210	0.04126	Heptadecanoyl carnitine
HMDB60065	0.042182	N-methyl-4,6,7-trihydroxy-1,2,3,4-tetrahydroisoquinoline
HMDB01138	0.048465	N-Acetylglutamic acid

Supplementary Figure 6: Statistically significant metabolites altered in C3(1)SV40Tag-p16-luc and EO771 mouse breast tumors. A. All metabolites with $p<0.05$ in C3(1)SV40Tag-p16-luc mouse breast tumors according to HMDB ID. **B**. All metabolites with $p<0.05$ in EO771 mouse breast tumors according to HMDB ID.

C3(1)SV40Tag-p16-luc

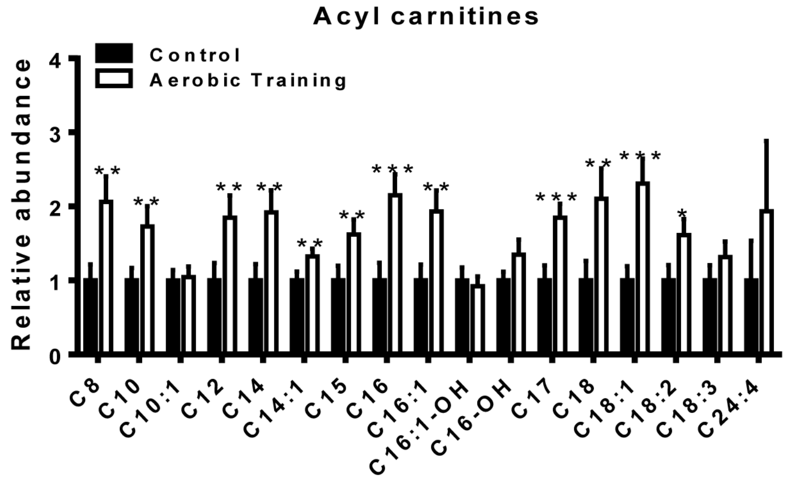

EO771

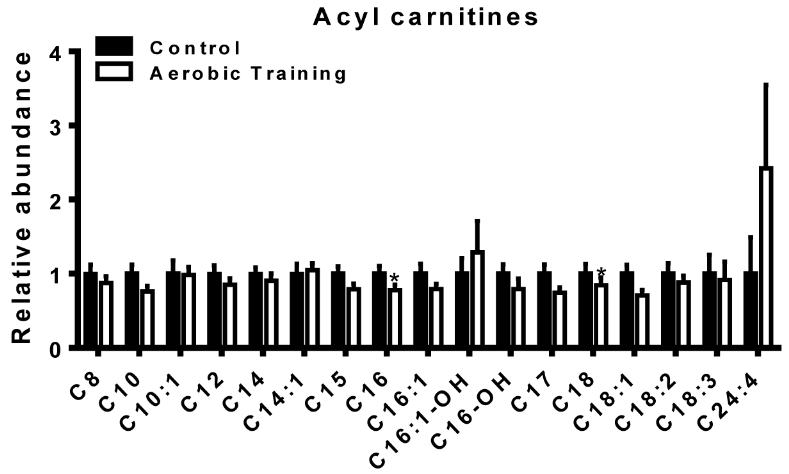

Supplementary Figure 7: Acyl carnitine metabolites in C3(1)SV40Tag-p16-luc and EO771 mouse breast tumors. (Top) C3(1)SV40Tag-p16-luc mouse tumors. The abundance of each metabolite is normalized to that in control group; mean + SEM. (Bottom) EO771 mouse tumors. The abundance of each metabolite is normalized to that in control group; mean + SEM.

Supplementary Table 1: Analysis of effect of exercise on tumor growth rates

Table 1A: Estimated effects for E0771 model

Term	Effect	Std.Error	t-value	p-value
**Baseline (μ)**	1.74	0.44	3.94	
**Initial effect (α_j)**	-0.28	0.49	-0.57	0.57
**Control rate (β)**	0.31	0.03	10.48	<0.0001
**Exercise effect (β_j)**	-0.01	0.03	-0.21	0.83

For the EO771 model, the growth rate in the control group was 31%/day (Table 1A), but there was no significant effect of exercise on the growth rate (p-value = 0.83).

Table 1B: Estimated effects for 4T07 model

Term	Effect	Std.Error	t-value	p-value
**Baseline (μ)**	3.27	0.39	8.46	
**Initial effect (α_j)**	-0.38	0.50	-0.77	0.44
**Control rate (β)**	0.16	0.03	5.73	<0.0001
**Exercise effect (β_j)**	0.03	0.04	0.91	0.37

For the 4T07 model, the growth rate in the control group was 16%/day (Table 1B), but there was no significant effect of exercise on the growth rate (p-value = 0.37).

Table 1C: Estimated effects for C3(1)SV40Tag-p16-luc model

Term	Effect	Std.Error	t-value	p-value
**Baseline (μ)**	3.13	0.53	5.89	
**Initial effect (α_j)**	-1.17	0.66	-1.78	0.08
**Control rate (β)**	0.20	0.05	4.16	<0.0001
**Exercise effect (β_j)**	0.08	0.06	1.43	0.15

For the C3(1)SV40Tag-p16-luc model, the growth rate in the control group was 20%/day (Table 1C), but there was no significant effect of exercise on the growth rate (p-value = 0.15).

Supplementary Table 2: Analysis of late effect of aerobic training on tumor growth rates

To identify if there were late changes to the growth rate, we also fit model (1.1) to data after day 13 of each mouse tumor model.

Table 2A: Estimated effects for late changes in EO771 model

Term	Effect	Std.Error	t-value	p-value
Baseline (μ)	0.74	0.90	0.82	
Initial effect (α_j)	1.55	1.17	1.32	0.19
Control rate (β)	0.37	0.06	6.64	<0.0001
Exercise effect (β_j)	-0.12	0.07	-1.62	0.11

For the EO771 model, the late change control growth rate of 37%/day (Table 2A) was similar to the mean growth rate in the control group of 31%/day (Table 1A). There was no significant effect of exercise on the late change growth rate (p-value = 0.11).

Table 2B: Estimated effects for late changes in 4T07 model

Term	Effect	Std.Error	t-value	p-value
Baseline (μ)	5.40	1.61	3.36	
Initial effect (α_j)	-0.22	2.00	-0.11	0.91
Control rate (β)	0.03	0.10	0.35	0.73
Exercise effect (β_j)	0.02	0.12	0.15	0.88

For the 4T07 model, the late change control growth rate of 3%/day (Table 2B) was considerably slower than the mean growth rate in the control group was 16%/day (Table 1B), but there was no significant effect of exercise on the late change growth rate (p-value = 0.88).

Table 2C: Estimated effects for late changes in C3(1)SV40Tag-p16-luc model

Term	Effect	Std.Error	t-value	p-value
Baseline (μ)	8.17	2.83	2.89	
Initial effect (α_j)	-6.13	3.11	-1.97	0.06
Control rate (β)	-0.16	0.20	-0.81	0.42
Exercise effect (β_j)	0.43	0.21	2.01	0.05

For the C3(1)SV40Tag-p16-luc model, the late change control growth rate of -16%/day (Table 2C) was considerably slower than the mean growth rate in the control group was 20%/day (Table 1C), The late change growth rate in the exercise group was faster, but the difference was marginally significant (p-value = 0.05).

Supplementary Table 3: Analysis of effect of aerobic training and digoxin on tumor volume

	Effect	Std. Error	t value	p-value
**Baseline**	5.64	0.18	31.96	
**Digoxin**	0.52	0.37	1.42	0.17
**Aerobic Training (AT)**	0.43	0.26	1.62	0.11
**Digoxin: AT**	-1.24	0.55	-2.24	0.03

ANOVA analysis of combined C3(1)Sv40Tag-p16-luc data sets with interaction effects.

We note that the main (monotherapy) effects of digoxin and aerobic training cause a slight increase in tumor volume relative to baseline, but these effects are not statistically significant. However the interaction effect (combination therapy) is significantly negative (p-value = 0.03), suggesting combining the therapies counteracts the small increases we would expect with the individual mono-therapies. It does not, however, mean a decrease relative to baseline.
